# Increased expression of Protein S in eyes with diabetic retinopathy and diabetic macular edema

**DOI:** 10.1038/s41598-021-89870-5

**Published:** 2021-05-17

**Authors:** Masahiko Sugimoto, Mineo Kondo, Taro Yasuma, Corina N. D’Alessandro-Gabazza, Masaaki Toda, Hisanori Imai, Makoto Nakamura, Esteban C. Gabazza

**Affiliations:** 1 Department of Ophthalmology, Mie University Graduate School of Medicine, 2-174, Edobashi, Tsu, Mie 514-8507 Japan; 2 Department of Immunology, Mie University Graduate School of Medicine, Tsu, Mie Japan; 3 Division of Ophthalmology, Department of Surgery, Kobe University Graduate School of Medicine, Kobe, Japan

**Keywords:** Eye diseases, Diabetes complications

## Abstract

Protein S (PS) is a multifunctional glycoprotein that ameliorates the detrimental effects of diabetes mellitus (DM). The aim of this study was to evaluate the distribution of PS in diabetic retinopathy (DR) and diabetic macular edema (DME). This was a study of 50 eyes with DM (37 with DME, 6 with proliferative DR, and 7 with no DR) and 19 eyes without DM. The level of PS was measured by enzyme immunoassay and was compared between eyes with or without DM, with or without DME, and with severe DME (≥ 350 μm) or mild DME (< 350 μm). We also performed immunohistopathologic evaluations of post-mortem eyes and the cystoid lesions excised during surgery. The aqueous free PS was significantly higher with DM (7.9 ± 1.2 ng/ml, *P* < 0.01) than without DM (6.1 ± 0.7). The aqueous free PS was significantly elevated with DME (8.2 ± 1.2, *P* < 0.05) compared to proliferative DR (7.0 ± 1.0) and no DR (7.0 ± 0.7). Eyes with severe DME had significantly higher aqueous free PS than mild DME (8.5 ± 1.3 vs. 7.7 ± 1.0, *P* < 0.05). Immunohistochemistry showed PS in the outer plexiform layer of the retina and cystoid lesion. The higher expression of PS with DR and DME suggests that PS is involved in their pathogenesis.

## Introduction

There are approximately 400 million people with diabetes mellitus (DM) worldwide, and it has been estimated that the number will reach 600 million in 20 years^1^. DM is characterized by the destruction of the pancreatic β cells leading to impaired insulin secretion, hyperglycemia, and chronic microangiopathy^2^. Common microvascular complications of DM include peripheral neuropathy, nephropathy, and retinopathy. Diabetic retinopathy (DR) causes severe visual disturbances due to retinal ischemia in eyes and an increase of retinal permeability leading to diabetic macular edema (DME)^3^. The retinal ischemia causes protein leakage and edema because of the disruption of the blood-retinal barrier between the retinal vascular endothelium and retinal pigment epithelium^4^. The prevalence of DME is different from that of DR and not all DM patients develop DME. An earlier study showed that among 22,896 DM patients, the overall prevalence of DR was 34.6% and that for DME was 6.81%^5^.

The angiogenic and inflammatory responses in eyes with DR play a critical role in the progression of DR and DME. The presence of inflammation has led to a growing interest in developing inhibitors of the inflammation to treat these retinal disorders. In this context, the pro-inflammatory and angiogenic vascular endothelial growth factor (VEGF) is an important therapeutic target, and anti-VEGF agents are currently the first-line treatment for DME. Inhibitors of inflammation, including steroids, e.g., triamcinolone acetonide, dexamethasone, and fluocinolone acetonide, are also used to treat non-responders of anti-VEGF agents. However, no curative therapy is currently available for DME. Therefore, identifying the mechanism-associated factors and developing biomarkers for the early detection of these disorers are of critical importance.

Protein S (PS) is a 75-kDa vitamin K-dependent glycoprotein that regulates inflammation by inhibiting the coagulation system, the expression of inflammatory cytokines from several types of cells, and apoptosis^6^. PS regulates inflammation and apoptosis by binding to the Tyro3, Axl, and Mer (TAM) tyrosine kinase receptors^7,8^. PS circulates in the plasma in a free form and can complex with C4b-binding protein (C4BP), an inhibitor of the classic complement pathway. Both the free form of PS (free PS) and complex form of PS exert anti-inflammatory activity. For example, PS in complex with C4BP inhibits complement-mediated inflammation by localizing C4BP to the cell membrane^9^. These reported beneficial effects suggest the potential therapeutic application of PS for inflammatory diseases^10^.

We have reported that PS is also protective in eyes of patients with DM^11^. Increased circulating free PS levels in transgenic mice inhibit apoptosis of pancreatic β cells and ameliorates DM, and the systemic administration of PS improves kidney fibrosis and renal dysfunction in diabetic mice^11^. The high frequency of vascular events, including deep vein thrombosis and pulmonary emboli in patients with PS deficiency highlights the clinical relevance of the anticoagulant function of PS^12^.

The maintenance of the homeostasis of the retinal vessel is another important role of PS. PS deficiency in a mouse model is associated with retinal changes resembling retinopathy of prematurity^13^. PS also regulates the phagocytosis in the outer layers of the retina and contributes to maintaining a normal retinal architecture^14^. However, the role of PS in diabetic retinal complications has not been examined.

Thus, it is important to assess the role played by PS in eyes with DR and DME. The purpose of this study is to evaluate distribution of PS in diabetic eyes as a first step to accomplish this. We measured the level of PS in the blood and aqueous humor. We also performed histological examination to determine the site of expression of PS in a diabetic retina.

## Results

### Demographics of patients

The demographics of the patients are shown in Table 1. Among the eyes with DM, 37 eyes had DME, 7 eyes had NDR, and 6 eyes had PDR. In the group with DME, 27 eyes had cystic DME, 9 eyes had sponge DME, and 1 eye had serus DME. The NDR group was significantly older than the other groups (*P* = 0.0003, Kruskal–Wallis test). The group with no DM had significantly lower fasting blood glucose than the other groups (*P* = 0.0001).

**Table 1:** Demographics of all patients.

N		Age	HbA1c	FBS	BUN	eGFR	DBP	SBP
*DM(* +*)*		(years)	(%)	(mg/dL)	(mg/dL)	(mL/min/1.73m^2^)	(mmHg)	(mmHg)
DME	37	65.3 ± 12.6	7.8 ± 1.6	173.3 ± 65.9	20.0 ± 12.9	65.9 ± 32.2	141.6 ± 20.5	73.0 ± 10.8
NDR	7	75.0 ± 5.9*	6.5 ± 0.3	135.1 ± 36.9	19.9 ± 6.9	72.2 ± 16.3	139.9 ± 17.6	71.3 ± 13.1
PDR	6	67.5 ± 10.4	7.9 ± 1.9	182.3 ± 34.3	21.2 ± 9.1	52.5 ± 27.5	140.3 ± 29.9	73.0 ± 12.3
Total	50	68.2 ± 10.0	7.6 ± 1.6	168.5 ± 58.9	20.1 ± 11.6	65.1 ± 29.7	141.1 ± 21.0	72.8 ± 11.1
*DM*( −)	19	73.5 ± 7.5	-	107.1 ± 15.4*	14.9 ± 3.2	70.3 ± 12.5	138.4 ± 15.6	78.1 ± 8.3
*P* value		0.0003*	0.07	0.0001*	0.79	0.86	0.94	0.56

BUN: blood urea nitrogen, DBP: diastolic blood pressure, DM: diabetes mellitus, DME: diabetic macular edema, eGFR: estimated glomerular filtration rate, FBS: fasting blood sugar, HbA1c: hemoglobin A1c, NDR: no diabetic retinopathy, PDR: proliferative diabetic retinopathy, SBP: systolic blood pressure.

*: *P* < 0.05, Kruskal–Wallis test.

### Plasma and aqueous humor levels of PS and related molecules in patients with and without DM

**Figure 1:**
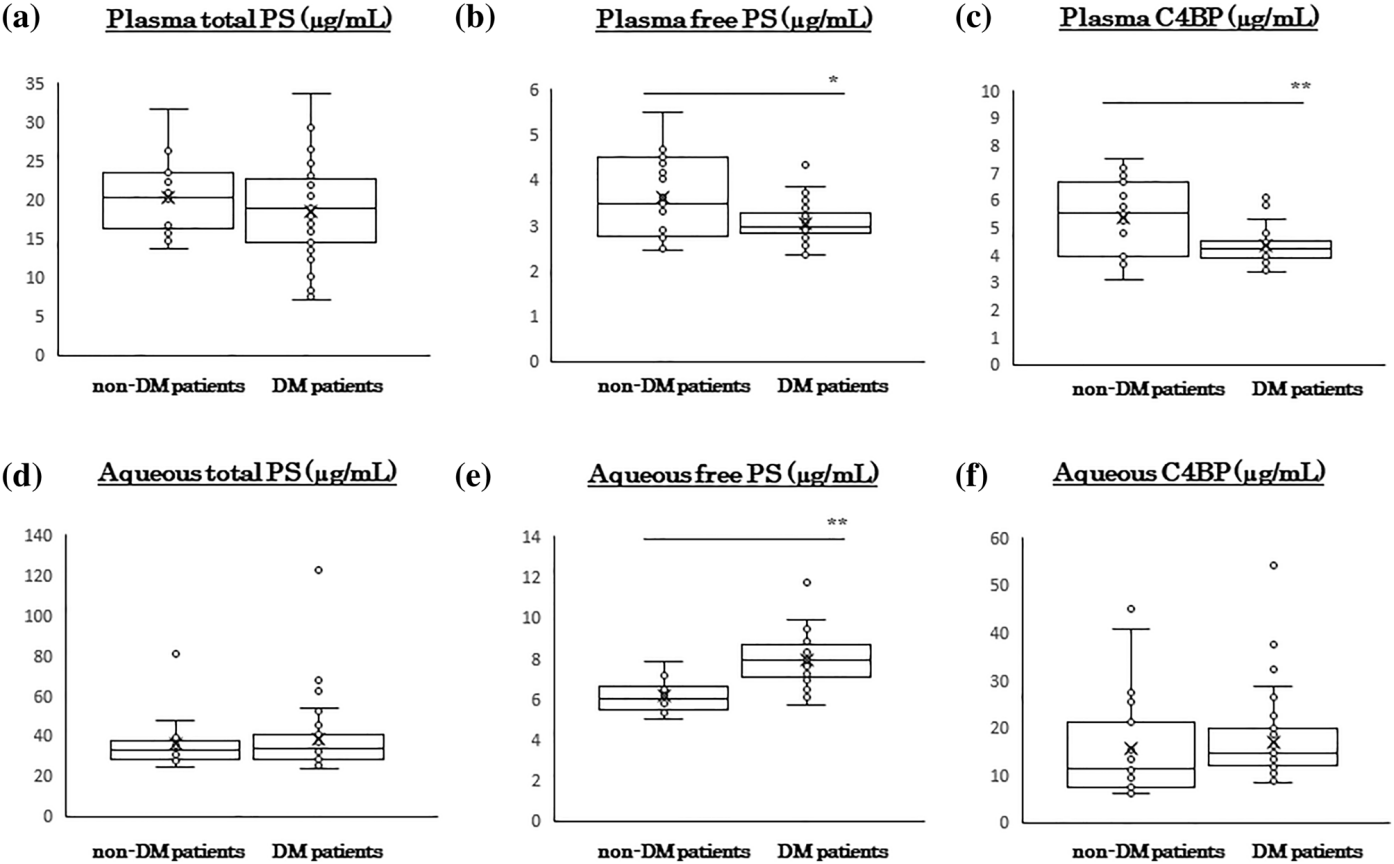
Plasma and aqueous humor concentrations of Protein S and related molecules in patients with or without diabetes mellitus (DM). The plasma concentration of total PS is not significantly different in the DM patients and non-DM patients (**a**). Plasma levels of free PS and C4BP are significantly lower in the DM patients than in the non-DM patients (**b**, **c**). The total PS and C4BP levels are not significantly different among the different groups (**d**, **f**). The free PS level in the aqueous humor is significantly higher in the DM patients than in the non-DM patients (**e**). C4BP, C4b-binding protein; DM, diabetes mellitus; PS, protein S. *P < 0.05, **P < 0.01. Statistical analyses by Mann–Whitney U test.

The concentration of total PS in the plasma of patients with DM was 18.5 ± 5.9 μg/ml which was not significantly different from that in patients without DM at 20.2 ± 4.5 μg/ml (*P* > 0.05). The concentration of PS in the aqueous humor of patients with DM was 38.6 ± 16.3 ng/ml which was not significantly different from that in patients without DM at 35.6 ± 12.2 ng/ml (*P* > 0.05; Fig. 1a,d). The plasma concentrations of free PS in patient with DM was 3.0 ± 0.4 μg/ml which was significantly lower than that in patients without DM at 3.6 ± 0.9 μg/ml (*P* = 0.01). The plasma concentration of C4BP was 4.3 ± 0.7 μg/ml in patients with DM which was significantly lower than the 5.4 ± 1.4 μg/ml in patients without DM (*P* = 0.01 Fig. 1b,c). The concentration of free PS in the aqueous humor was 7.9 ± 1.2 ng/ml in patients with DM which was significantly higher than that in non-DM patients at 6.1 ± 0.7 ng/ml (*P* = 6.20 × 10^−9^; Fig. 1e). There was no significant difference in the concentration of C4BP in the aqueous humor between the DM group (17.1 ± 8.2 ng/ml) and non-DM group (15.8 ± 11.4 ng/ml; Fig. 1f).

### Plasma and aqueous humor levels of PS and related molecules in DME, non-DR, and PDR groups

**Figure 2:**
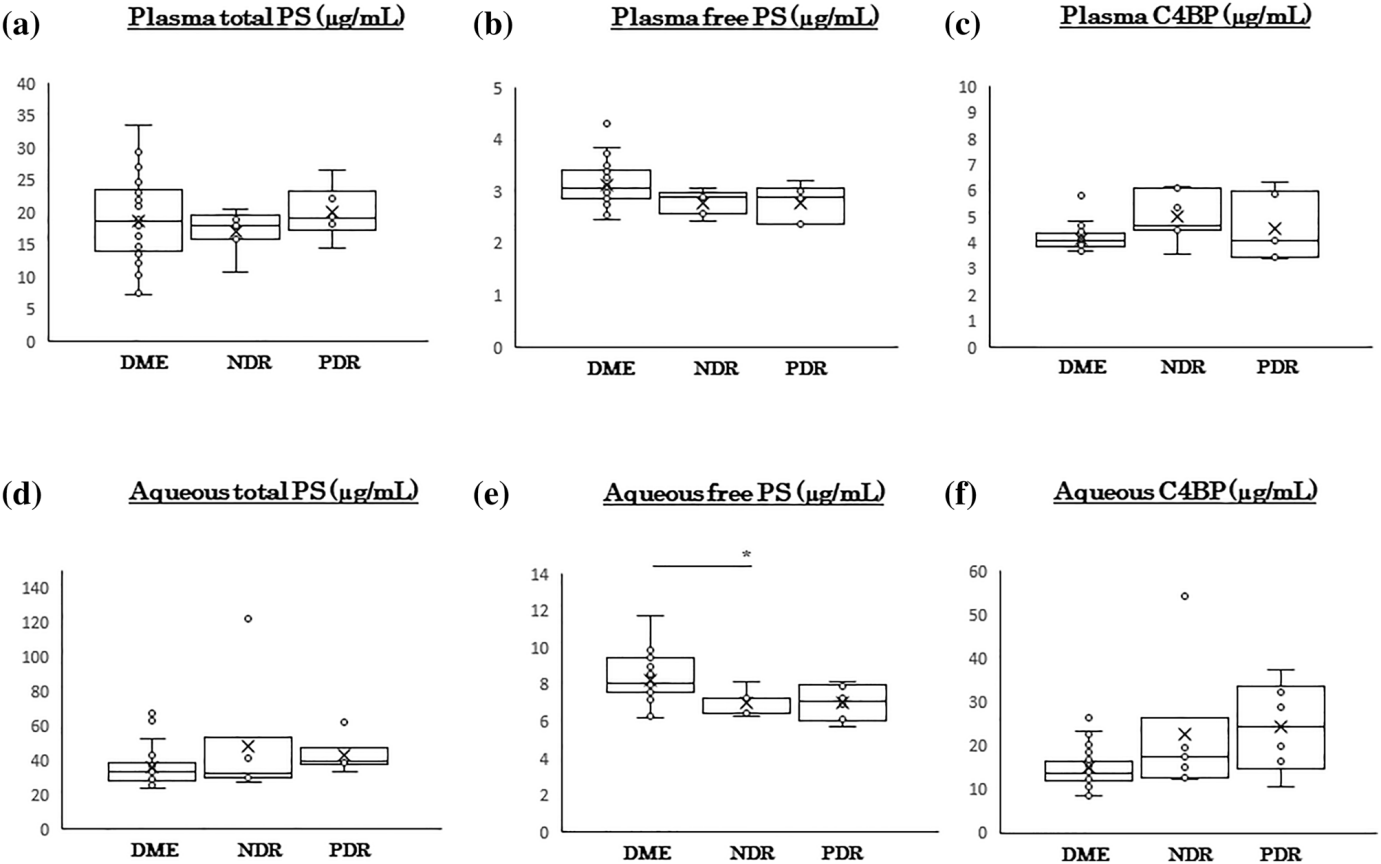
Plasma and aqueous humor concentrations of protein S and related molecules in patients with diabetic retinopathy (DR). The plasma levels of total PS (**a**), free PS (**b**), and C4BP (**c**) are not significantly different among the different groups. The aqueous humor levels of total PS and C4BP are also not significantly different (**d**, **f**). The aqueous humor level of free PS is significantly higher in the DME group than the other groups (**e**). C4BP, C4b-binding protein; DM, diabetes mellitus; PS, protein S. *P < 0.05. Statistical analysis by Kruskal–Wallis test.

There was no significant difference in the plasma concentrations of total PS (18.6 ± 6.5 μg/ml vs 17.1 ± 3.2 μg/ml vs 19.9 ± 4.1 μg/ml), free PS (3.1 ± 0.4 μg/ml vs 2.8 ± 0.2 μg/ml vs 2.8 ± 0.4 μg/ml). and C4BP (4.2 ± 0.4 μg/ml vs 5.0 ± 0.9 μg/ml vs 4.5 ± 1.3 μg/ml) among the DME, NDR and PDR groups (Fig. 2a–c). There was also no significant difference in the aqueous humor concentrations of total PS (36.1 ± 11.2 ng/ml vs 48.3 ± 33.8 ng/ml vs 42.8 ± 10.0 ng/ml), and C4BP (14.8 ± 4.4 ng/ml vs 22.5 ± 14.7 ng/ml vs 24.1 ± 10.3 ng/ml) among the three groups (Fig. 2d,f). The concentration of free PS in the aqueous humor was 8.2 ± 1.2 ng/ml in the DME group which was significantly higher than the 7.0 ± 0.7 ng/ml in the NDR group and the 7.0 ± 1.0 ng/ml in the PDR group (*P* = 6.78 × 10^−7^; Fig. 2e).

### Severity of DME and ocular PS concentration

**Figure 3:**
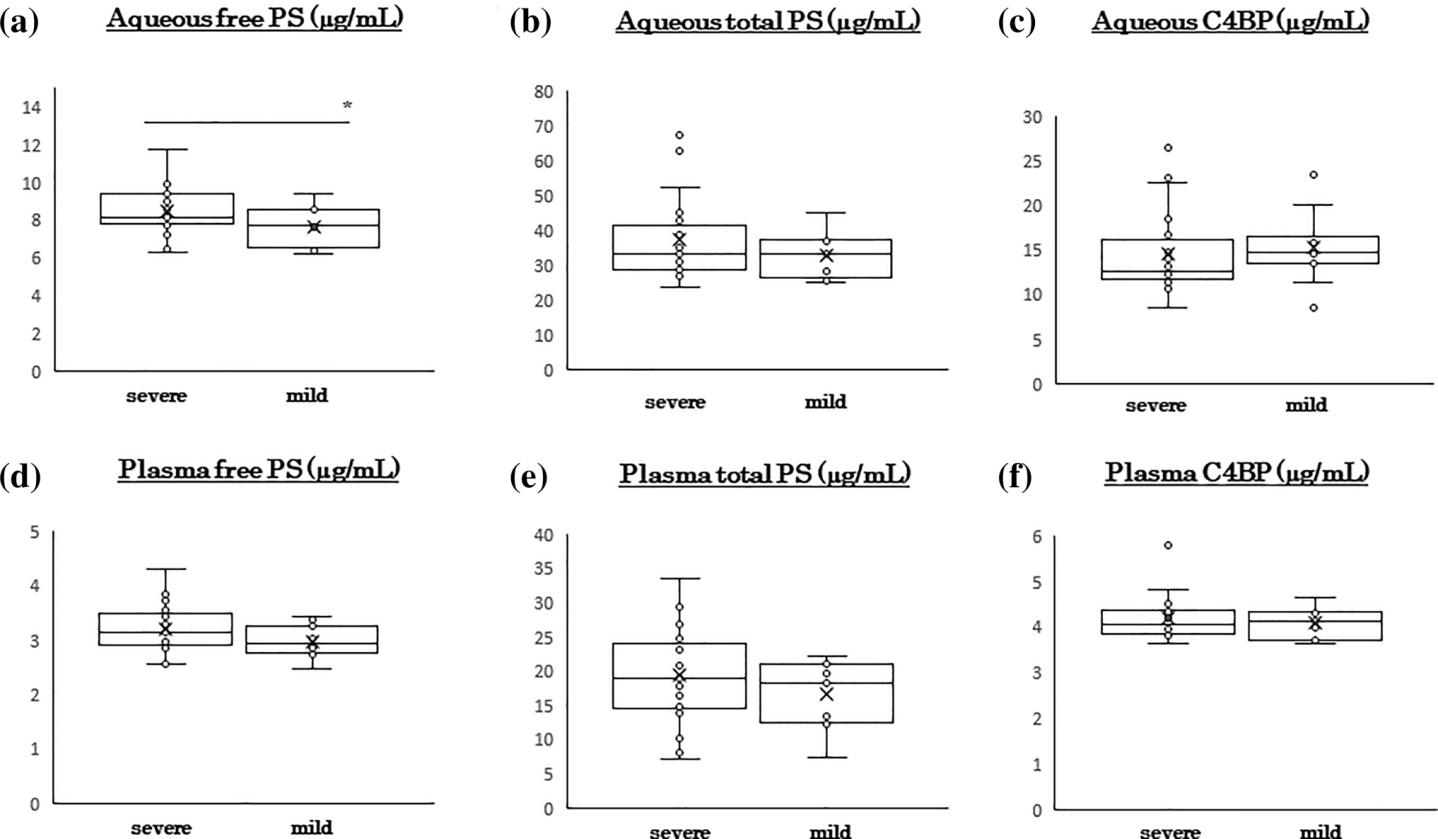
Degree of DME affects ocular protein S concentration. The DME eyes were divided into two groups; severe DME group (CMT ≥ 350 μm, n = 25) and mild DME group (CMT < 350 μm, n = 12). The aqueous humor free PS level is significantly higher in the severe DME group than in the mild DME group (**a**). The aqueous humor levels of total PS (**b**) and C4BP (**c**) are not significantly different between the groups. The plasma levels of free PS (**d**), total PS (**e**), and C4BP (**f**) were not significantly different between the groups. C4BP, C4b-binding protein; CMT, central subfoveal macular thickness; DM, diabetes mellitus; DME, diabetic macular edema; PS, protein S. *P < 0.05. Statistical analysis by Mann–Whitney U test.

Because there was a high concentration of free PS in the aqueous humor in the DME group, we divided the eyes with DME into two groups: a group with severe DME (CRT ≥ 350 μm, n = 25) and a group with mild DME (CRT < 350 μm, n = 12). The demographics of the patients are shown in Table 2. The values of the different parameters were not significantly different between the two groups other than CMT. The aqueous humor level of free PS was significantly higher in the group with severe DME than the group with mild DME (8.5 ± 1.3 ng/ml vs 7.7 ± 1.0 ng/ml, *P* = 0.04, Fig. 3a). The aqueous humor concentrations of total PS (37.6 ± 12.6 ng/ml vs 32.7 ± 6.6 ng/ml) and C4BP (14.6 ± 4.6 ng/ml vs15.4 ± 4.0 ng/ml) were not significantly different between patients with severe and mild DME (Fig. 3b,c). The plasma concentrations of free PS (3.2 ± 0.4 μg/ml vs 3.0 ± 0.3 μg/ml), total PS (19.5 ± 7.1 μg/ml vs 16.5 ± 4.8 μg/ml), and C4BP (4.2 ± 0.5 μg/ml vs 4.1 ± 0.3 μg/ml) were not significantly different (Fig. 3d–f) between the two groups.

**Table 2:** Demographics of the DME patients.

N		Age	CMT	HbA1c	FBS	BUN	eGFR	DBP	SBP
		(years)	(μm)	(%)	(mg/dL)	(mg/dL)	(mL/min/1.73m^2^)	(mmHg)	(mmHg)
High (≧350 μm)	25	64.1 ± 12.2	473.3 ± 83.6*	7.9 ± 1.8	181.0 ± 35.9	20.7 ± 15.1	69.6 ± 32.5	142.2 ± 21.5	73.7 ± 11.3
Low (< 350 μm)	12	68.3 ± 13.8	309.6 ± 42.1	7.7 ± 1.2	157.2 ± 66.5	18.2 ± 5.2	57.8 ± 31.6	140.1 ± 18.8	71.6 ± 10.1
*P* value		0.40	5.80 × 10^−5^*	0.82	0.36	0.62	0.35	0.78	0.62

BUN: blood urea nitrogen, CMT: central subfoveal macular thickness, DBP: diastolic blood pressure, eGFR: estimated glomerular filtration rate, FBS: fasting blood sugar, HbA1c: hemoglobin A1c, SBP: systolic blood pressure.

### Localization of PS in human retina

**Figure 4:**
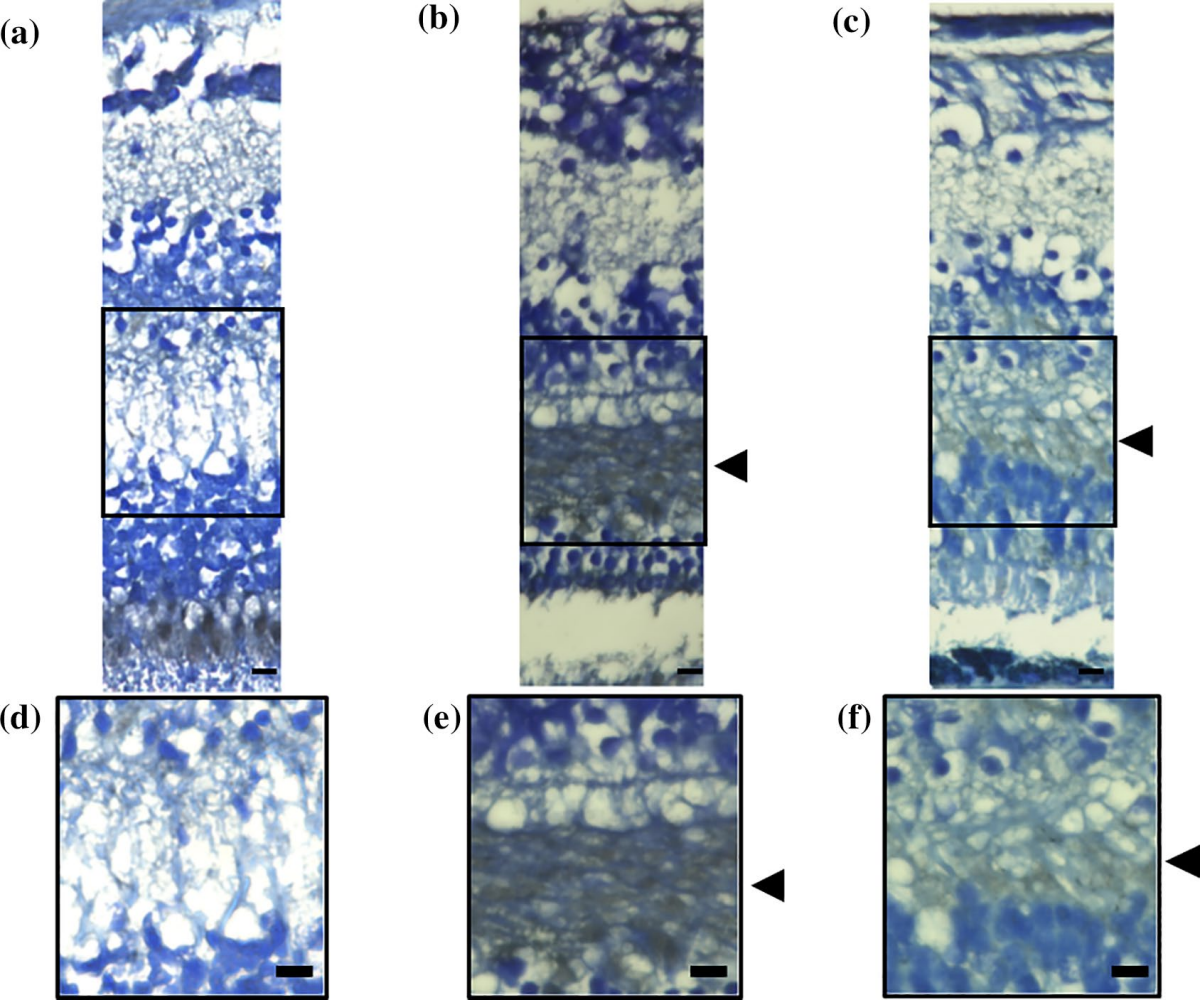
Location of protein S in the human retina with and without diabetes. The retina of post-mortem human eyes was stained with a PS antibody. No staining was observed in the retina from patients without DM (62-years-old, woman, (**a**). PS is present in the OPL of patients with DM (**b**, 93-year-old, man with NPDR; 60-year-old, man with PDR). Black arrow heads shows localization of PS on OPL. Magnified images of each images were shown (**d**–**f**). DM, diabetes mellitus; NPDR, non-proliferative diabetic retinopathy; OPL, outer plexiform layer; PDR, proliferative diabetic retinopathy; PS, protein S. Bar: 10 μm.

We stained the retina of post-mortem human eyes to determine the expression sites of PS in eyes with DM. The retina of the eyes from a non-DM patient (62-years-old, woman; Fig. 4a,d) did not have any localized staining for PS. However, PS staining was positive in the outer plexiform layer (OPL) of the diabetic eyes of a 93-year-old man with NPDR (Fig. 4b,e) and a 60 years-old man with PDR (Fig. 4c,f).

### PS location in cystoid lesion in eyes with DME

**Figure 5:**
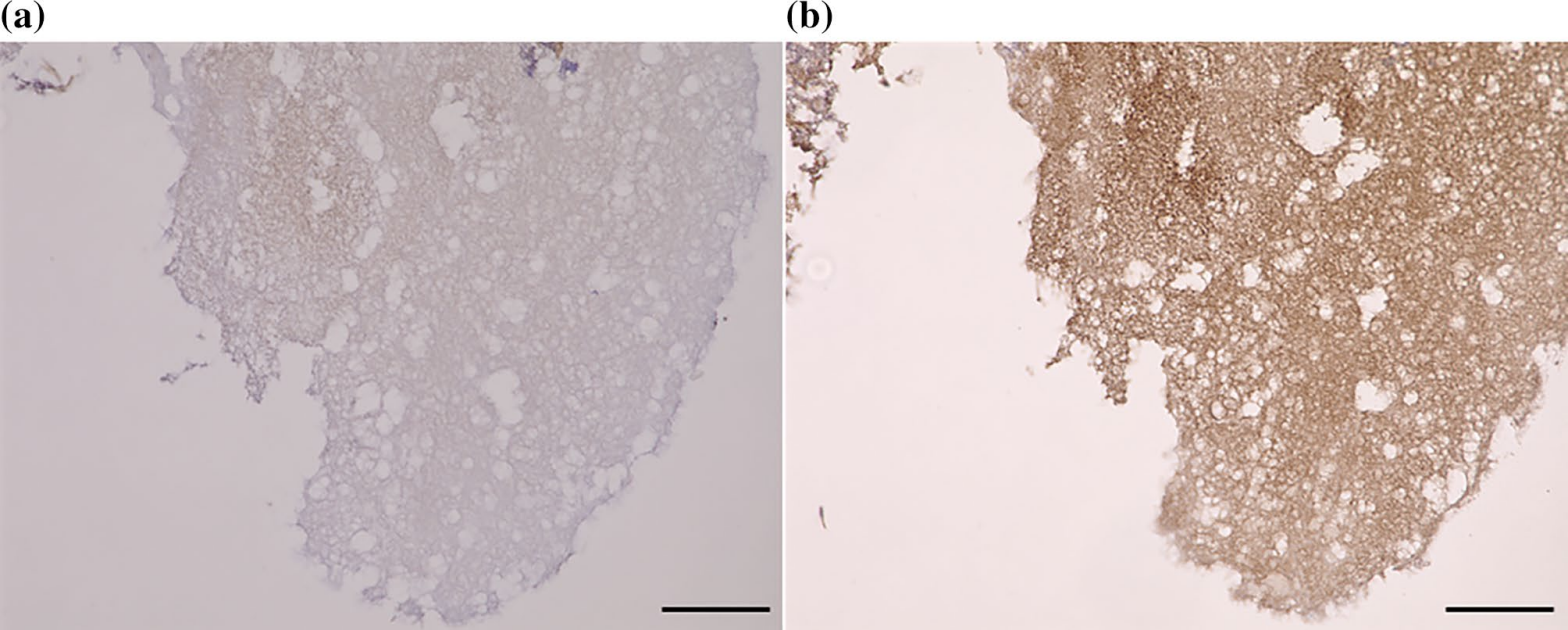
Location of Protein S in the components of a cystoid lesion with diabetic macular edema. The component of cystoid lesion with DME was stained with anti-hPS antibody. No staining was observed in the control nonspecific IgG (**a**). PS was identified inside the component of cystoid lesion (**b**). DME, diabetic macular edema; PS, protein-S. Bar: 100 μm.

We collected specimens of the cystoid lesions for PS staining in eyes with DME during pars plana vitrectomy. The specimens stained positive for PS (Fig. 5a,b).

## Discussion

Macular edema is the accumulation of extravascular fluid in the OPL, the inner nuclear layer, and the Müller cells leading to localized expansion^15^. Cystoid macular edema has a perifoveal configuration with cyst-like spaces due to fluid accumulation^16,17^. The presence of cystic changes in eyes with DME in the OPL is common by pathological examinations^18^. Byeon et al. also reported cysts within the outer plexiform and OPL that may be related to leaky vessels when examined by OCT^19^. The cysts frequently progress and become hard exudates in the outer plexiform and outer nuclear layers. Deposits of the hard exudates causes irreversible retinal damages leading to loss of central visual function. Thus, the evidence suggests that the outer plexiform and outer nuclear layers are major sites of tissue injury in DME. Our findings showed an increase of free PS in DM eyes particularly in severe DME, localized expression of PS in the OPL, and within cysts in cystic DME. These findings suggest the close association of PS expression with the DME pathological conditions.

PS inhibits the coagulation system by increasing the activity of activated protein C (APC), an anticoagulant protein^20^. PS can accelerate fibrinolysis by suppressing the activation of thrombin-activatable fibrinolysis inhibitor or by supporting the APC-mediated inhibition of plasminogen activator inhibitor-1^21^. Fibrinogen is a soluble macro-molecule that forms an insoluble clot or gel after conversion to fibrin^22^. The C4BP-PS complex may accelerate fibrin degradation during inflammation by interacting with plasminogen^23^. This increased interaction of C4BP and PS with plasminogen accelerates the plasminogen activator-mediated conversion of plasminogen to plasmin on the fibrin surface which leads to increased fibrinolysis. Mass spectrometry analysis has shown that the components of the cystoid lesion in eyes with DME is composed of microfibrin wrapped in collagen fibrils and fibrinogen^24^. Based on these observations, we suggest that the PS present in the OPL and inside the cystoid lesion activates the fibrinolysis pathway to reduce the deposition of fibrin. This effect may also prevent hard exudates deposition that causes irreversible retinal damage. The results of earlier studies have shown that PS protects the blood–brain barrier from hypoxic/ischemic damage through its Tyro3 receptor, and mice deficient in PS develop embryonic lethal coagulopathy with disruption of the blood–brain barrier^25,26^. Based on these observations, we can speculate that PS protects against blood-retinal barrier disruption which contributes significantly to protein leakage with DME and it may explain our observation, significant higher the aqueous humor levels of free PS level in the DME group compared to the NPDR and PDR groups. But because small number of samples were evaluate PS levels in PDR and NPDR for ELISA which was not balanced, this may affect the results. In addition, we could obtain a few number of post-mortem eye with DM because of the difficulty to obtain post-mortem eye for research in Japan. We need further investigation with many number of tissue samples.

An interesting observation was the discrepant levels of free PS in plasma and aqueous humor. While there was decreased levels of free PS in plasma, the free PS level was increased in the aqueous humor. Synthesis and release of PS by cells in the eyes may explain these findings. However, although vascular endothelial cells can secrete PS, there is no evidence showing the expression of PS by retinal capillary endothelial cells. The biological significance of the elevated level of PS in the eyes with DM is also unclear. Although the local increase of PS in the eyes may be a compensatory response to an enhanced retinal injury, a detrimental role of PS in disease pathogenesis also deserves consideration. Such dissociation of the molecule between local and systemic distribution is sometimes observed for other molecules. Though many cytokines are expressed in skeletal muscle following exercise, some of them are not released into the circulation at least in large amounts because they are produced locally and sufficient quantities of cytokines to increase their concentration in the systemic circulation may not be secreted^27^. But because our histological examination couldnot certify the origin of the PS expression, it is not cleaer whether similar spiculation can explain dissociation of local and systemic PS distribution. We need much consideration for this matter in the further study.

On the contraly, there were several reports about unfavorable effects of PS. PS exacerbates acute liver injury by prolonging the natural killer T cells survival and worsens liver fibrosis by inhibiting apoptosis of extracellular matrix-producing fibroblasts^28,29^. The expression of PS may also be disease stage-related. Zhong et al. have reported a high glomerular level of PS in early stages and low glomerular PS level in late stages of diabetic nephropathy despite the normal circulating level of PS^30^. There is a possibility that PS distribution may differ among the stage of disease. However, the occurrence of DME is not related to the clinical stage of DR^5^. Based on our present findings, we suggest that PS expression is not related to the stage of DR and that PS expression only increases in DME. Therefore, detecting PS expression may be a biomarker for the early diagnosis of DME before the abnormalities are detected by OCT or ophthalmoscopy. However, further investigations are needed to corroborate these findings.

Though we reported importance of PS in diabetic retina, there are some limitations in this study including the small number of samples. First, the inclusion of a larger number of eyes with cyst-type DME than sponge- or serus-type DME are limitations of the present study. There is a possibility that PS behavior differ with other type of DME like sponge or serus type. The relationship between the retinal morphologic changes and concentrations of intravitreal cytokines in eyes with DME was reported and the significant association of serus type DME with intravitreal interleukin-6 level was observed and which indicated that inflammation may play an important role in the development of serus type DME^31^. Because there is a possibility PS may only affect the formation of cystic DME, we also need advanced examination for various DME type.

Second, the circadian rhythm is important for in the progression of arteriosclerosis and thrombosis^32^. Undar et al.^33^. reported that plasma PS levels were also affected by circadian rhythm, significantly the highest at 6 a.m. and the lowest at noon. We did not take into account physiological chage of PS due to circadian variation. But they reported that no significant differences were observed at other hours and intervals. Though we collectted samples at daily time (from 9 a.m to 5 p.m) when PS levels were not reported to be affected^33^, we should take into account the effects of circadian rhythm.

And finally, Here we did not evaluate vitreous level of PS for DR and DME. There is a possibility that PS levels in vitreous fluid reflects PS levels in retina more sensitive. But some reports concern that there is a significant relationship between VEGF and interleukin-6 levels in aqueous humor and in vitreous fluid^34^. Though it is not clear whether this is also applied for PS, we need consideration for this in the further study.

Our results showed the presence and increased expression of PS in eyes with DR and DME. The expression of PS was especially high in the OPL and cystoid lesions. These observations suggest that PS is involved in the pathogenesis of diabetic retinal complications, and it can be used as a biomarker and therapy for DME.

## Methods

### Subjects

The Mie University Ethics Committee for Clinical Investigations approved the investigation protocol (Approval #3087) and Kobe University Ethics Committee for Clinical Investigations approved the investigation protocol (Approval #B200233). The study was registered at http://www.umin.ac.jp (UMIN ID 000033728). The procedures conformed to the tenets of the Declaration of Helsinki, and all patients signed an informed consent form before entry. The patients enrolled were patients in the Department of Ophthalmology and Endocrinology, Mie University Hospital and Kobe University. The clinical history of all patients was obtained from their medical records.

### Sample collection

Blood serum samples were collected during regular checkups before receiving any ophthalmic treatments. The samples were centrifuged 400 × g for 5 min, the supernatants removed, and immediately stored at − 80 °C until use. In addition, 50 μl of aqueous humor was collected before the ocular treatments. The samples were frozen immediately and stored at − 80 °C until use. We did not collect any vitreous fluid because sampling can cause complications such as retinal detachments.

### Inclusion and exclusion criteria

Patients that were ≥ 20-years with type 1 or type 2 DM and those with DR or DME were included in the DM group. The diagnosis of DME was based on findings of the fundus examinations and spectral-domain optical coherence tomography (OCT). The stage of diabetic nephropathy before treatment was obtained from the medical charts. Trained retinal specialists (M.S. and M.K.) examined the fundus by indirect ophthalmoscopy and wide field fundus imaging obtained by the Optos ultra-widefield imaging system (Optos Panoramic 200MA™, Optos PLC. Dunfermline, Scotland, UK). The severity of DR was classified into three groups from these evaluations: no DR (NDR), non-proliferative DR (NPDR), and proliferative DR (PDR) according to the International Clinical Diabetic Retinopathy Disease Severity Scale (DRSS)^35^. DME was defined as a central subfoveal macular thickness (CMT) of ≥ 250 μm measured as the mean retinal thickness in the central 1 mm diameter circle in the OCT images. Subjects with NDR or NPDR had no DME.

The exclusion criteria for patients with DM were; history of any pars plana vitrectomy, history of intravitreal or sub-tenon injections of any drugs including anti-VEGF agents within two months before the beginning of this study, eyes with any inflammatory disease, drusen, vitreous hemorrhage or retinal hemorrhage which involved the intra- or subfoveal spaces, glaucoma or intraocular pressure ≥ 21 mmHg, and media opacities that significantly affected the OCT images. Women with pregnancy, under fertility treatments or use of contraceptives were also excluded from all the groups.

We also collected aqueous humor from patients without DM during surgical procedures for cataract, macular hole, and retinal detachment. The inclusion criteria for patients without DM were; patients ≥ 20-years with no DM and IOP of ≤ 21 mmHg. The exclusion criteria for patients without DM were; patients with severe systemic disorders, any history of other ocular diseases, history of pars plana vitrectomy, or glaucoma. DME eyes were also divided into two groups based on CMT measurement: a group with severe DME (higher CMT more than 350 μm) and a group with mild DME (lower CMT less than 350 μm).

The exclusion criteria for both DM patients and non-DM control subjects were; uncontrolled systemic medical conditions, history of a thromboembolic event or ischemic disease including myocardial infarction or cerebral infarction, prior treatment with anticoagulants, i.e., aspirin or with systemic anti-VEGF agents, i.e., bevacizumab, diagnosis of diseases causing hypercoagulability, high refractive errors (spherical equivalent >  − 3 or + 3 diopters, axial lengths longer than 24.0 mm or less than 22.0 mm, and amblyopia.

Hematological analyses were done before the treatment to measure the level of fasting blood sugar (FBS, normal value 70–110 mg/dL), hemoglobin A1c (HbA1c, NGSP, normal value 4.9–6.0%), blood urea nitrogen (BUN, normal value 8–20 mg/dL), and estimated glomerular filtration rate (eGFR, normal value 60–120 ml/min/1.73 m^2^). The blood pressure was also measured before beginning the treatment.

### Immunoassays

To determine the level of free human PS (hPS), a 96-well microplate was coated with C4BP (ATGen Corp., Sampyeong-dong, Korea), and after appropriate washing and blocking, samples were incubated to allow free hPS to bind C4BP. Then, biotin-labeled polyclonal rabbit anti-hPS antibody (Dako Cytomation, Glostrup, Denmark) was added. Complement C4BP was measured using an enzyme immunoassay kit from Assaypro (St. Charles, MO) and the total hPS as described^28^.

### Immunohistochemical examination of human eyes

Post-mortem human eyes were obtained from the National Disease Research Interchange (Philadelphia, PA) fixed in formalin and embedded in paraffin. The postmortem time ranged from 14 to 24 h. The slides were deparaffinized in xylene and rehydrated through an alcohol series for staining. Endogenous peroxidase was blocked by immersion in 0.3% hydrogen peroxidase for 30 min. Slides were incubated with goat polyclonal anti-hPS antibody (A0384, DAKO, Glostrup, Denmark) followed by treatment with horseradish peroxidase. Samples were examined with a fluorescence microscope (BZ-9000: Keyence, Osaka, Japan).

### *En bloc* removal of the component of cystoid lesion

The components of cystoid lesions in eyes with DME were collected during pars plana vitrectomy. The surgical procedure for *en bloc* extraction of cystoid lesion component combined with pars plana vitrectomy in DME patients was described in detail^24^. Briefly, after incision of the external wall of the subfoveal cystoids with a 27-gauge instruments, the exposed components of the cystoid lesions were grasped by forceps and excised *en bloc*. After removal, the cystoid lesion components were stored at − 80 °C and embedded in paraffin at the time of examination.

### Statistical analyses

Data are presented as the means ± standard deviations. The significance of the differences between two variables was determined by Mann–Whitney U-tests and between three or more variables by the Kruskal–Wallis test with the Scheffe test. A *P* < 0.05 was considered significant.

## References

[1] Wild S (2004). Global prevalence of diabetes: estimates for the year 2000 and projections for 2030. Diabetes Care.

[2] American Diabetes Association (2015). Classification and diagnosis of diabetes. Diabetes Care.

[3] Klein R, Klein BE, Moss SE (1984). Visual impairment in diabetes. Ophthalmology.

[4] Finkelstein D (1992). Ischemic macular edema. Recognition and favorable natural history in branch vein occlusion. Arch. Ophthalmol..

[5] Yau JW (2012). Global prevalence and major risk factors of diabetic retinopathy. Diabetes Care.

[6] Ahnstrom J, Andersson HM, Canis K (2011). Activated protein C cofactor function of protein S: a novel role for a gamma-carboxyglutamic acid residue. Blood.

[7] Hafizi S, Dahlback B (2006). Gas6 and protein S. Vitamin K-dependent ligands for the Axl receptor tyrosine kinase subfamily. FEBS J..

[8] Linger RM, Keating AK, Earp HS, Graham DK (2008). TAM receptor tyrosine kinases: biologic functions, signaling, and potential therapeutic targeting in human cancer. Adv. Cancer Res..

[9] Bouwens EA, Stavenuiter F, Mosnier LO (2013). Mechanisms of anticoagulant and cytoprotective actions of the protein C pathway. J. Thromb. Haemost..

[10] Rezende SM, Simmonds RE, Lane DA (2004). Coagulation, inflammation, and apoptosis: different roles for protein S and the protein S-C4b binding protein complex. Blood.

[11] Yasuma T (2016). Amelioration of diabetes by protein S. Diabetes.

[12] Comp PC, Nixon RR, Cooper MR, Esmon CT (1984). Familial protein S deficiency is associated with recurrent thrombosis. J. Clin. Investig..

[13] Burstyn-Cohen T, Heeb MJ, Lemke G (2009). Lack of protein S in mice causes embryonic lethal coagulopathy and vascular dysgenesis. J. Clin. Investig..

[14] Hall MO (2005). Both protein S and Gas6 stimulate outer segment phagocytosis by cultured rat retinal pigment epithelial cells. Exp. Eye Res..

[15] Scholl S, Kirchhof J, Augustin AJ (2010). Pathophysiology of macular edema. Ophthalmologica.

[16] Wolter JR (1981). The histopathology of cystoid macular edema. Albrecht Von Graefes Arch. Klin. Exp. Ophthalmol..

[17] Rotsos TG, Moschos MM (2008). Cystoid macular edema. Clin. Ophthalmol..

[18] Tso MO (1982). Pathology of cystoid macular edema. Ophthalmology.

[19] Byeon SH (2012). New insights into the pathoanatomy of diabetic macular edema: angiographic patterns and optical coherence tomography. Retina.

[20] Dahlback B (2018). Vitamin K-Dependent Protein S: Beyond the Protein C Pathway. Semin. Thromb. Hemost..

[21] Mosnier LO, Meijers JC, Bouma BN (2001). The role of protein S in the activation of thrombin activatable fibrinolysis inhibitor (TAFI) and regulation of fibrinolysis. Thromb. Haemost..

[22] Weisel JW, Litvinov RI (2017). Fibrin formation, structure and properties. Subcell Biochem..

[23] Agarwal V, Talens S, Grandits AM, Blom AM (2015). A novel interaction between complement inhibitor C4b-binding protein and plasminogen that enhances plasminogen activation. J. Biol. Chem..

[24] Imai H (2020). Effectiveness of en bloc removal of fibrinogen-rich component of cystoid lesion for the treatment of cystoid macular edema. Retina.

[25] Saller F (2009). Generation and phenotypic analysis of protein S-deficient mice. Blood.

[26] Zhu D (2010). Protein S controls hypoxic/ischemic blood-brain barrier disruption through the TAM receptor Tyro3 and sphingosine 1-phosphate receptor. Blood.

[27] Peake JM, Gatta PG, Suzuki K, Nieman DC (2015). Cytokine expression and secretion by skeletal muscle cells: regulatory mechanisms and exercise effects. Exerc Immunol Rev..

[28] Chelakkot-Govindalayathil AL (2015). Protein S exacerbates alcoholic hepatitis by stimulating liver natural killer T cells. J. Thromb. Haemost..

[29] Totoki T (2018). Protein S exacerbates chronic liver injury and fibrosis. Am. J. Pathol..

[30] Zhong F (2018). Protein S protects against podocyte injury in diabetic nephropathy. J. Am. Soc. Nephrol..

[31] Sonoda S (2014). Retinal morphologic changes and concentrations of cytokines in eyes with diabetic macular edema. Retina.

[32] Man AWC, Li H, Xia N (2021). Circadian rhythm: potential therapeutic target for atherosclerosis and thrombosis. Int. J. Mol. Sci..

[33] Undar L, Ertuğrul C, Altunbaş H, Akça S (1999). Circadian variations in natural coagulation inhibitors protein C, protein S and antithrombin in healthy men: a possible association with interleukin-6. Thromb. Haemost..

[34] Funatsu H (2005). Aqueous humor levels of cytokines are related to vitreous levels and progression of diabetic retinopathy in diabetic patients. Graefes Arch. Clin. Exp. Ophthalmol..

[35] Wilkinson CP (2003). Proposed international clinical diabetic retinopathy and diabetic macular edema disease severity scales. Ophthalmology.

